# Silybin Alleviated Hepatic Injury by Regulating Redox Balance, Inflammatory Response, and Mitochondrial Function in Weaned Piglets under Paraquat-Induced Oxidative Stress

**DOI:** 10.3390/antiox13030324

**Published:** 2024-03-06

**Authors:** Long Cai, Dongxu Ming, Wenning Chen, Ying Zhao, Yanpin Li, Wenjuan Sun, Yu Pi, Xianren Jiang, Xilong Li

**Affiliations:** 1 Key Laboratory of Feed Biotechnology of the Ministry of Agriculture and Rural Affairs, Institute of Feed Research, Chinese Academy of Agricultural Sciences, Beijing 100081, China; 82101211211@caas.cn (L.C.); mdx920825@163.com (D.M.); 82101215363@caas.cn (W.C.); ying.zhao@student.uliege.be (Y.Z.); liyanpin@caas.cn (Y.L.); sunwenjuan@caas.cn (W.S.); piyu@caas.cn (Y.P.); 2 Precision Livestock and Nutrition Unit, TERRA Teaching and Research Centre, Gembloux Agro-Bio Tech University of Liege, 5030 Gembloux, Belgium

**Keywords:** silybin, hepatic damage, inflammation, antioxidation, mitochondrial function, apoptosis, weaned piglets

## Abstract

Silybin (Si) is the main element of silymarin isolated from the seeds of *Silybum marianum* L. Gaernt., which has superior antioxidant properties. However, the protective role of Si in maintaining liver health under oxidative stress remains ambiguous. This study aimed to investigate the underlying mechanism of the beneficial effect of dietary Si against hepatic oxidative injury induced by paraquat (PQ) in weaned piglets. A total of 24 piglets were randomly allocated to four treatments with six replicates per treatment and 1 piglet per replicate: the control group; Si group; PQ group; and Si + PQ group. Piglets in the control group and PQ group were given a basal diet, while piglets in the Si and Si + PQ groups were given a Si-supplemented diet. On the 18th day, the pigs in the PQ treatment group received an intraperitoneal injection of PQ, and the others were intraperitoneally injected with the same volume of saline. All piglets were sacrificed on day 21 for plasma and liver sample collection. The results showed that dietary Si supplementation mitigated PQ-induced liver damage, as proven by the reduction in liver pathological changes and plasma activity of alanine transaminase and aspartate transaminase. Si also improved superoxide dismutase and glutathione peroxidase activities and total antioxidant capacity, as well as decreased malondialdehyde and hydrogen peroxide concentration in the liver, which were closely related to the activation of the nuclear factor-erythroid 2-related factor 2 signaling pathway. Meanwhile, Si reduced tumor necrosis factor-α and interleukin-8 production and their transcript levels as well as abrogated the overactivation of nuclear factor-κB induced by PQ. Importantly, Si improved mitochondrial function by maintaining mitochondrial energetics and mitochondrial dynamics, which was indicated by the elevated activity of mitochondrial complexes I and V and adenosine triphosphate content, decreased expression of dynamin 1 protein, and increased expression of mitofusin 2 protein. Moreover, Si inhibited excessive hepatic apoptosis by regulating the B-cell lymphoma-2 (Bcl-2)/Bcl-2-associated-X-protein signaling pathway. Taken together, these results indicated that Si potentially mitigated PQ-induced hepatic oxidative insults by improving antioxidant capacity and mitochondrial function and inhibiting inflammation and cell apoptosis in weaned piglets.

## 1. Introduction

The liver is the principal organ responsible for metabolism and detoxification. It plays a crucial role in nutrient storage, xenobiotic metabolism, antioxidation, and immunoregulation within the body [1]. However, young animals are susceptible to liver injury induced by various stressors, including dietary and environmental stress, due to their physiological immaturity [2]. Oxidative stress is recognized as a vital pathological mechanism underlying the initiation and progression of liver injury [3]. Excessive reactive oxygen species (ROS) induce lipid peroxidation, protein oxidation, and DNA damage [4,5,6]. More importantly, free radicals interfere with the process of mitochondrial dynamics and bioenergetics, and then cause mitochondrial structure and function disorders [7], subsequently aggravating liver intracellular oxidative stress and triggering endogenous apoptotic pathways, ultimately leading to liver injury and dysfunction [8,9]. Therefore, it is necessary to develop effective nutrition intervention strategies to protect animals against liver damage injury from oxidative stress.

In recent years, significant attempts have been made to improve liver health through dietary antioxidants such as phytonutrients. Silybin, as a kind of flavonoid lignan, is the main active constituent of silymarin extracted from milk thistle, and plays an essential role in eliminating ROS and regulating antioxidant capacity [10]. In addition, silybin possesses a wide range of pharmacological activities, such as hepatoprotective, anti-inflammatory, and antibacterial activities [11,12], and improves mitochondrial function by regulating biogenesis and bioenergetics [13,14]. Dietary silymarin/silybin has been reported to improve growth performance and exert a promoting effect on systemic health in pigs and poultries [15,16,17,18]. Consistently, our previous research also found that dietary silybin decreased diarrhea incidence and improved growth performance in weaned piglets; moreover, silybin supplementation effectively alleviated intestinal injury by improving the antioxidant properties, mitochondrial function, and microbial community composition of weaned piglets [19]. However, the beneficial effects of silybin on liver health in weaned piglets under oxidative stress, and their underlying mechanisms, are still unknown.

Therefore, the purpose of this present study was to explore the potential of silybin in attenuating liver oxidative injury challenged with paraquat (PQ). PQ is a well-known redox imbalance inducer that can produce ROS continuously by interfering with the electron transmission on the mitochondrial respiratory chain, thereby inducing damage to different organs, including the liver, gastrointestinal tract, and other organs [20,21,22,23]. Thus, it is widely used to establish an oxidative stress model [24,25]. We measured the effect of dietary silybin on liver pathological changes, plasma biochemical parameters, hepatic enzyme activity, and inflammatory cytokine content. Subsequently, the activation of the Nrf2/Keap1 and NF-κB signaling pathways, mitochondrial function-related gene and protein expression, and initiation of hepatocytic apoptosis were further analyzed. Our findings can provide a novel nutritional intervention strategy for improving the liver health of weaned piglets and lay a theoretical foundation for the incorporation and utilization of silybin in animal feed.

## 2. Materials and Methods

### 2.1. Animal Ethics Approval

All animal procedures in this study complied with the ARRIVE guidelines and were approved by the Animal Care and Use Committee of the Institute of Feed Research of the Chinese Academy of Agricultural Sciences (IFR-CAAS20220428) [26].

### 2.2. Animals and Treatment

A total of 24 Duroc × (Landrace × Yorkshire) weanling piglets of similar age (28 ± 1 d) and initial body weight (7.68 ± 0.37 kg) were randomly allocated to 4 treatments with 6 replicates (pens) per treatment, ensuring an equal distribution of initial body weight and sex (half male and half female). The treatment included the Ctrl group (basal diet); Si group (basal diet supplemented with 400 mg/kg silybin); PQ group (basal diet); and Si + PQ group (basal diet supplemented with 400 mg/kg silybin). On the 18th day, the pigs in the PQ group and Si + PQ group received an intraperitoneal injection of 4 mg/kg BW paraquat (PQ) (methyl viologen hydrate, Huaxia Chemical Reagent Co., Ltd., Chengdu, China), and other groups received an intraperitoneal injection of saline. The dose of PQ to induce oxidative stress in the liver of the piglets was determined according to a previous report [25]. The administered level of silybin (purity > 97%, Panjin Tianyuan Pharmaceutical Co., Ltd., Panjin, China) in this study was determined based on a previous study [19]. The experiment was conducted at the Tianpeng experimental farm and lasted for a total of 21 days. The ingredient and nutrient levels of the basal diet met the nutritional requirements according to the National Research Council (2012) and have been described in our previous report [19,27]. Piglets were given ad libitum access to feed and fresh water in pens with slatted floors.

### 2.3. Sample Collection

At the end of the trial (day 21), approximately 6 mL blood samples were collected via the jugular veins of the piglets and centrifuged at 3000× *g* for 4 °C at 10 min to obtain plasma. Subsequently, all piglets were euthanized after being stunned by a portable electrical stunner (the output voltage was 220 V). The liver samples from the right medial lobe were collected and snap-frozen in liquid nitrogen and stored at −80 °C for further analysis. In addition, portions of the liver samples from the right medial lobe were fixed in fresh 4% paraformaldehyde for hematoxylin and eosin (H&E) staining analysis.

### 2.4. Plasma Biochemical Analysis

The activities of alanine transaminase (ALT), aspartate transaminase (AST), and alkaline phosphatase (ALP) in plasma were measured using a corresponding commercial kit (Jiangsu Meimian Industrial Co., Ltd., Yancheng, China) as described by the manufacturer’s instructions.

### 2.5. Determination of Inflammatory Cytokine Content

The frozen liver was homogenized in chilled saline using an automatic homogenizer. Then, the homogenate was centrifuged at 3000× *g* for 15 min at 4 °C to obtain the supernatant, and the total protein concentration in the liver supernatant was subsequently detected. The content of tumor necrosis factor-α (TNF-α) and interleukin (IL)-6, IL-8, and IL-10 in the liver homogenate were measured using porcine-specific enzyme-linked immunoassay kits (Shanghai Enzyme-linked Biotechnology Co., Ltd., Shanghai, China) according to the manufacturer’s protocols.

### 2.6. Determination of Enzyme Activity

Following the instructions of the protocols, the activities of superoxide dismutase (SOD), catalase (CAT), and glutathione peroxidase (GSH-Px); the total antioxidant capacity (T-AOC) level; and the content of malondialdehyde (MDA) and hydrogen peroxide (H_2_O_2_) in the liver supernatant were detected using kits purchased from Nanjing Jiancheng Biotechnology Co., Ltd. (Nanjing, China). The activities of mitochondrial complex I (COX I) and complex V (COX V) in the supernatant were measured using commercially available kits (Beijing Solarbio Science & Technology Co., Ltd., Beijing, China). After a similar homogenate step, the activity of caspase 3 and caspase 9 in the supernatant was determined using a specific kit purchased from Beyotime Biochem. Co., Ltd. (Shanghai, China). The assay steps were performed according to the instructions provided by the manufacturer.

### 2.7. Adenosine Triphosphate Content Assay

The liver homogenate was boiled on an electric hot plate for 10 min, cooled down on ice, and subsequently centrifuged at 3500× *g* for 10 min at 4 °C to obtain the supernatant. The content of adenosine triphosphate (ATP) in the supernatant was determined by phosphomolybdate colorimetry using a commercial kit (Nanjing Jiancheng Bioengineering Institute, Nanjing, China).

### 2.8. Histopathological Staining

The liver tissues were fixed with fresh 4% paraformaldehyde for more than 24 h, then embedded in paraffin after dehydration. The slices were stained with hematoxylin and eosin (H&E) for histopathological examination with an upright optical microscope (Niko, Tokyo, Japan). The histological score was assessed as described in a previous report [28].

### 2.9. Real-Time Quantitative PCR Analysis (RT-qPCR)

RNA extraction, reverse transcription, and qPCR of liver tissues were performed according to the procedure described in a previous report [29]. Briefly, total RNA was isolated using Trizol reagent (Thermo Fisher Scientific, Inc., Boston, MA, USA) according to the manufacturer’s instructions. Then, 1 μg RNA was used to produce cDNA by reverse transcription using the PrimeScript RT reagent kit (Takara Biotechnology Co., Ltd., Dalian, China). The real-time quantitative PCR was conducted using SYBR Green reagent (Thermo Fisher Scientific, MA, USA) on a CFX96 Touch real-time PCR instrument (Bio-Rad Laboratories Inc., Berkeley, CA, USA). The mRNA expression of the target gene relative to the housekeeping gene (*GAPDH*) was calculated using the 2^−ΔΔCT^ method. The primer sequences used in this study are listed in Table 1.

### 2.10. Western Blotting Analysis

The protein from liver tissues was extracted using an ice-cold RIPA buffer supplement with 1% phosphatase and protease inhibitors (Huaxing Biotechnology, Beijing, China). The total protein concentration of the extracting solution was measured with the BCA assay kit (Huaxing Biotechnology, Beijing, China). Then, the expression of the target proteins was detected by immunoblotting assays, and the relative abundance of protein expression was normalized to GAPDH. The specific antibodies were used as follows: Nrf2 (1:1000, Abcam, Cambridge, UK), Keap1 (1:1000, Abcam, Cambridge, UK), NF-κB p65 (1:1000, Cell Signaling Technology, Boston, MA, USA), Phospho-NF-κB p65 (1:500, Cell Signaling Technology, Boston, MA, USA), Drp1 (1:1000, Affinity Biosciences, Cincinnati, OH, USA), Mfn2 (1:1000, Affinity Biosciences, Cincinnati, OH, USA), Cleaved caspase 3 (1:1000, Affinity Biosciences, Cincinnati, OH, USA), Bax (1:1000, Affinity Biosciences, Cincinnati, OH, USA), Bcl-2 (1:1000, Abcam, Cambridge, UK), GAPDH (1:2000, Cell Signaling Technology, Boston, MA, USA), HRP-linked anti-rabbit IgG (1:2000, Cell Signaling Technology, Boston, MA, USA), and HRP-linked anti-mouse IgG (1:2000, Cell Signaling Technology, Boston, MA, USA).

### 2.11. Statistical Analysis

The data analysis was conducted by two-way ANOVA with SPSS statistical software version 19 (IBM, Armonk, NY, USA). One-way ANOVA was adopted to analyze the differences among all groups when the interaction was significant, followed by Tukey’s honest significant difference test for multiple comparisons. All data are expressed as mean with standard error (SE). A significance level of *p* < 0.05 was considered statistically significant, while a significance level of 0.05 ≤ *p* < 0.1 indicated a significant trend.

## 3. Results

### 3.1. Effects of Dietary Silybin Supplementation on Liver Injury in Piglets Challenged with PQ

**Figure 1:**
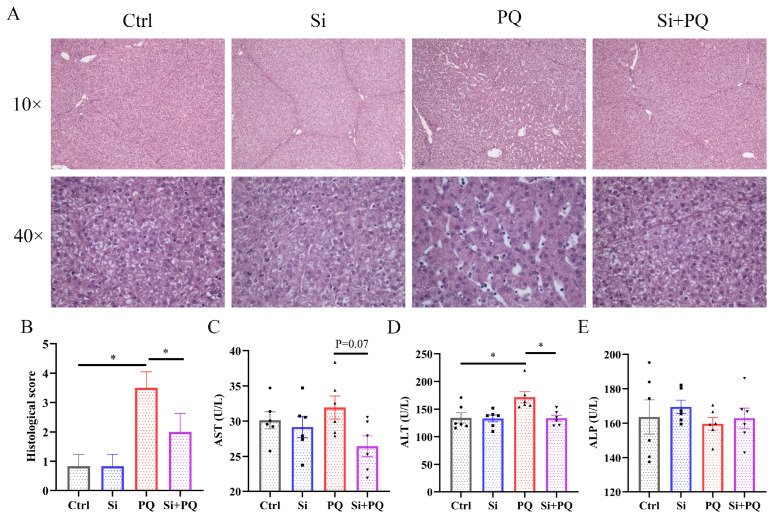
Dietary silybin supplementation alleviated liver injury induced by paraquat in piglets. (**A**) Representative H&E staining of liver tissues (captured at 100× or 400× magnification). (**B**) Histopathology scores of liver tissues. (**C**–**E**) Plasma activities of AST, ALT, and ALP. Ctrl, piglets were given a basal diet and were challenged with saline; Si, piglets were given a silybin-supplemented diet and were challenged with saline; PQ, piglets were given a basal diet and were challenged with paraquat; Si + PQ, piglets were given a silybin-supplemented diet and were challenged with paraquat; AST, aspartate transaminase; ALT, alanine transaminase; ALP, alkaline phosphatase. Data are expressed as mean ± standard error (n = 6). * *p* < 0.05.

As shown in Figure 1A, H&E staining showed that PQ challenge led to swelling and vacuolar degeneration of liver cells in the piglets, along with hepatic karyopyknosis and karyolysis. Conversely, dietary silybin supplementation alleviated the abovementioned pathological changes. Consistently, compared with the PQ group, dietary silybin administration significantly decreased the histological score of liver lesions in the PQ-challenged piglets (*p* < 0.05) (Figure 1B). Then, we further measured the activities of AST, ALT, and ALP in plasma and found that PQ challenge significantly increased ALT activity (*p* < 0.05). However, dietary silybin supplementation obviously reduced ALT activity (*p* < 0.05) and tended to decrease AST activity (*p* = 0.07) in the PQ-challenged piglets (Figure 1C,D). In addition, neither diet nor PQ treatment affected ALP activity in the plasma of the piglets (*p* > 0.05) (Figure 1E).

### 3.2. Effects of Dietary Silybin on Hepatic Oxidative Stress in Piglets Challenged with PQ

**Figure 2:**
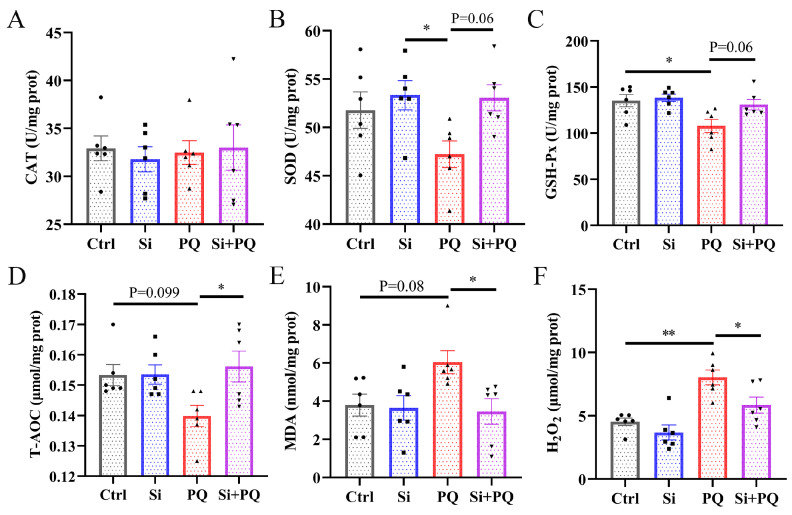
Dietary silybin administration ameliorated PQ-induced oxidative stress in the liver of piglets. The activities of CAT (**A**), SOD (**B**), and GSH-Px (**C**) and the level of T-AOC (**D**), MDA content (**E**), and H_2_O_2_ level (**F**) in the liver. Ctrl, piglets were given a basal diet and were challenged with saline; Si, piglets were given a silybin-supplemented diet and were challenged with saline; PQ, piglets were given a basal diet and were challenged with paraquat; Si + PQ, piglets were given a silybin-supplemented diet and were challenged with paraquat; CAT, catalase; SOD, superoxide dismutase; GSH-Px, glutathione peroxidase; T-AOC, the total antioxidant capacity; MDA, malondialdehyde; H_2_O_2_, hydrogen peroxide. Data are expressed as mean ± standard error (n = 6). * *p* < 0.05, ** *p* < 0.01.

As shown in Figure 2, compared with the control group, the piglets challenged with PQ had significantly decreased GSH-Px activity (*p* < 0.05) and increased H_2_O_2_ levels (*p* < 0.05), and tended to have a lower T-AOC level (*p* = 0.10) and higher MDA content (*p* = 0.08) (Figure 2C–E). In contrast, dietary silybin significantly increased the T-AOC level and decreased the concentration of MDA and H_2_O_2_ (*p* < 0.05), and tended to increase the activities of SOD (*p* = 0.06) as well as GSH-Px (*p* = 0.06) (Figure 2B–E). Moreover, there was no significant difference in CAT activity among all groups (*p* > 0.05) (Figure 2A).

### 3.3. Effects of Dietary Silybin on Hepatic Activation of Nrf2 Signaling Pathway in Piglets Challenged with PQ

**Figure 3:**
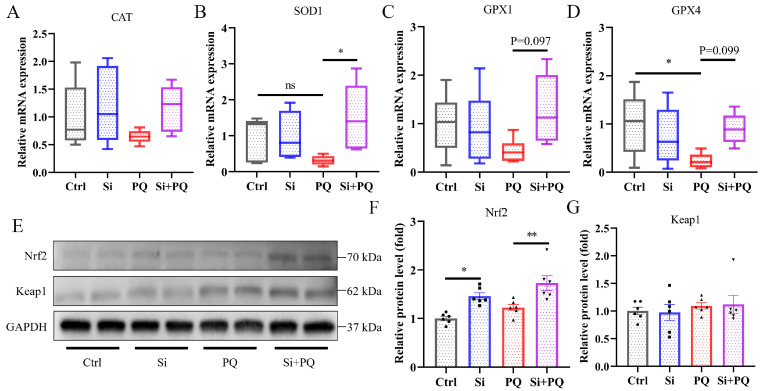
Silybin enhanced hepatic antioxidant capacity by activating the Nrf2 signaling pathway. The expression of Nrf2 signaling pathway genes including *CAT* (**A**), *SOD1* (**B**), *GPX1* (**C**), and *GPX4* (**D**). (**E**–**G**) The relative protein expression level of Nrf2 and Keap1. Ctrl, piglets were given a basal diet and were challenged with saline; Si, piglets were given a silybin-supplemented diet and were challenged with saline; PQ, piglets were given a basal diet and were challenged with paraquat; Si + PQ, piglets were given a silybin-supplemented diet and were challenged with paraquat; *CAT*, catalase; *SOD*, superoxide dismutase; *GPx*, glutathione peroxidase; *Nrf2*, nuclear factor-erythroid 2-related factor 2; *Keap1*, kelch-like ECH-associated protein l. Data are expressed as mean ± standard error (n = 6). * *p* < 0.05, ** *p* < 0.01.

As shown in Figure 3, we found that PQ challenge significantly decreased the expression level of the *GPX4* gene (*p* < 0.05), but not the expression levels of the *SOD1* and *GPX1* genes (*p* > 0.05) (Figure 3B–D). However, dietary silybin supplementation significantly elevated the mRNA expression of *SOD1* (*p* < 0.05) and tended to enhance the expression levels of *GPX1* (*p* = 0.097) and *GPX4* (*p* = 0.099) in the liver of the PQ-challenged piglets (Figure 3B–D). Neither silybin supplementation nor PQ treatment affected *CAT* mRNA expression in the liver (*p* > 0.05) (Figure 3A). Interestingly, the Western blotting results showed that silybin supplementation increased the nuclear factor-erythroid 2-related factor 2 (Nrf2) protein expression in the presence or absence of PQ challenge (*p* < 0.05) (Figure 3E–G).

### 3.4. Effects of Dietary Silybin on Inflammatory Response in Piglets Challenged with PQ

**Figure 4:**
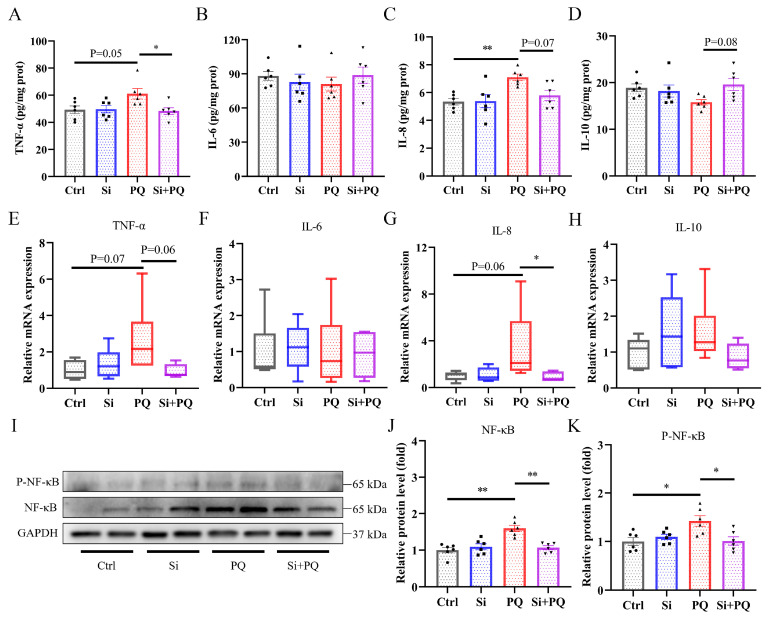
Dietary silybin supplementation alleviated hepatic inflammation induced by paraquat in piglets. The concentration of TNF-α (**A**), IL-6 (**B**), IL-8 (**C**), and IL-10 (**D**). The mRNA expression level of *TNF-α* (**E**), *IL-6* (**F**), *IL-8* (**G**), and *IL-10* (**H**). (**I**–**K**) The relative protein expression level of P-NF-κB and NF-κB. Ctrl, piglets were given a basal diet and were challenged with saline; Si, piglets were given a silybin-supplemented diet and were challenged with saline; PQ, piglets were given a basal diet and were challenged with paraquat; Si + PQ, piglets were given a silybin-supplemented diet and were challenged with paraquat; TNF-α, tumor necrosis factor-α; IL, interleukin; NF-κB, nuclear factor-kB; P-NF-κB, phosphorylated NF-κB. Data are expressed as mean ± standard error (n = 6). * *p* < 0.05, ** *p* < 0.01.

As shown in Figure 4, compared with the control group, PQ challenge caused an increase in the levels of TNF-α and IL-8 (*p* = 0.05 and *p* < 0.05, respectively) (Figure 4A,C). In contrast to the PQ group, silybin dramatically reversed these trends and increased IL-10 content in the liver (*p* = 0.08) (Figure 4A,C,D). Consistently, the relative mRNA abundance of *TNF-α* and *IL-8* was upregulated by PQ treatment (*p* = 0.07 and *p* = 0.06, respectively) (Figure 4E,G), compared with the control group. However, it was dramatically downregulated by dietary silybin supplementation (*p* = 0.06 and *p* < 0.05, respectively). In addition, dietary silybin significantly reduced the increase in protein abundance of nuclear factor-kB (NF-κB) and phosphorylated NF-κB induced by PQ treatment (*p* < 0.05) (Figure 4I,J).

### 3.5. Effects of Dietary Silybin on Mitochondrial Function in Piglets Challenged with PQ

**Figure 5:**
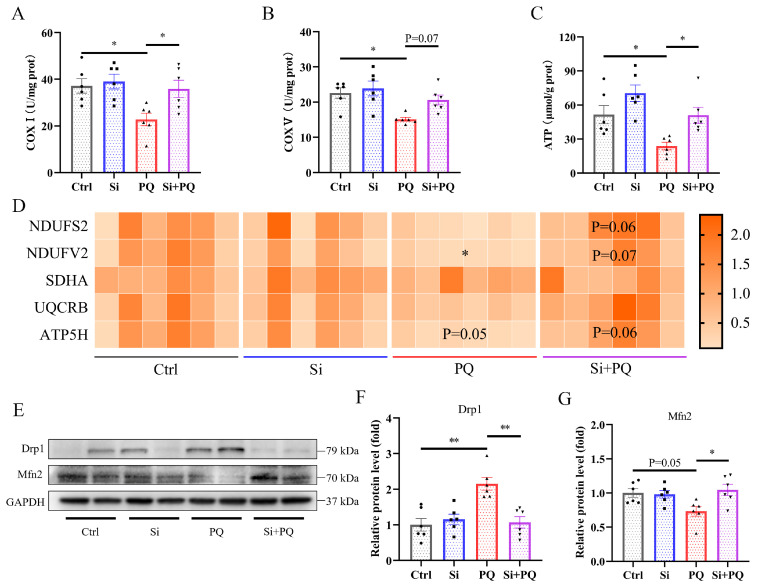
Dietary silybin supplementation protected against PQ-induced mitochondrial dysfunction. (**A**,**B**) The activities of COX Ⅰ and COX Ⅴ in the liver. (**C**) The level of ATP. (**D**) The expression of mitochondrial respiratory chain protein complex genes including *NDUFS2*, *NDUFV2*, *SDHA*, *UQCRB*, and *ATP5H*. (**E**–**G**) The relative protein expression level of Drp1 and Mfn2. Ctrl, piglets were given a basal diet and were challenged with saline; Si, piglets were given a silybin-supplemented diet and were challenged with saline; PQ, piglets were given a basal diet and were challenged with paraquat; Si + PQ, piglets were given a silybin-supplemented diet and were challenged with paraquat; COX, mitochondrial complex; ATP, adenosine triphosphate. *NDUFS2*, NADH ubiquinone oxidoreductase core subunit S2; *NDUFV2*, NADH ubiquinone oxidoreductase core subunit V2; *SDHA*, succinate dehydrogenase complex flavoprotein subunit A; *UQCRB*, ubiquinol-cytochrome c reductase binding protein; *ATP5H*, ATP synthase subunit d; Drp1, dynamin 1; Mfn2, mitofusin 2. Data are expressed as mean ± standard error (n = 6). * *p* < 0.05, ** *p* < 0.01.

As shown in Figure 5, PQ challenge decreased the activities of mitochondrial complexes I and V and subsequently reduced the ATP content in the liver (*p* < 0.05) (Figure 5A–C). However, dietary silybin supplementation alleviated the PQ-induced decrease in mitochondrial complex I and V activity compared to the PQ group, thereby enhancing the production of ATP (*p* < 0.05). Consistently, we found that PQ treatment downregulated the expression level of NADH ubiquinone oxidoreductase core subunit V2 (*NDUFV2*) (*p* < 0.05) and tended to decrease ATP synthase subunit d (*ATP5H*) (*p* = 0.05) compared to the control group (Figure 5D), while silybin supplementation elevated the abovementioned gene expression in the liver of the PQ-challenged piglets (*p* = 0.07 and *p* = 0.06, respectively). In addition, dietary silybin also upregulated the mRNA expression of *NADH* ubiquinone oxidoreductase core subunit S2 (*NDUFS2*) in response to PQ challenge (*p* = 0.06) (Figure 5D). Moreover, PQ challenge increased the protein abundance of dynamin 1 (Drp1) and decreased the protein abundance of mitofusin 2 (Mfn2), while silybin supplementation significantly reversed the abovementioned trend (*p* < 0.05) (Figure 5E–G).

### 3.6. Effects of Dietary Silybin Supplementation on Hepatocyte Apoptosis in Piglets Challenged with PQ

**Figure 6:**
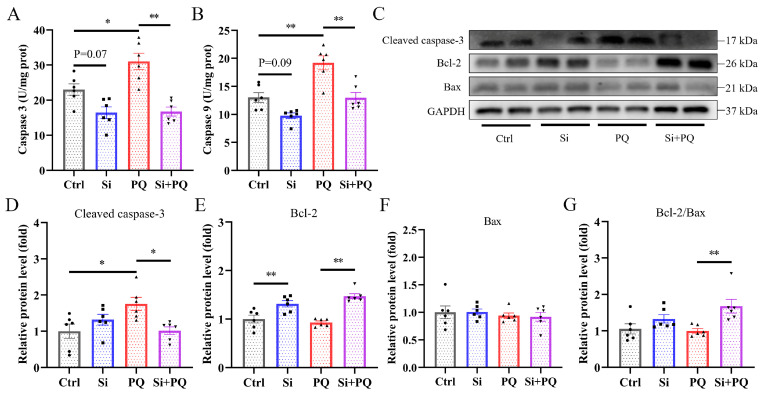
Dietary silybin addition suppressed hepatocyte apoptosis. (**A**,**B**) The activities of caspase 3 and caspase 9 in the liver tissues. (**C**) Representative image of Western blot. The protein expression levels of Cleaved caspase 3 (**D**), Bcl-2 (**E**), Bax (**F**), and the ratio of Bcl-2 to Bax (**G**). Ctrl, piglets were given a basal diet and were challenged with saline; Si, piglets were given a silybin-supplemented diet and were challenged with saline; PQ, piglets were given a basal diet and were challenged with paraquat; Si + PQ, piglets were given a silybin-supplemented diet and were challenged with paraquat; Bcl-2, B-cell lymphoma-2; Bax, Bcl-2-associated-X-protein; Bcl-2/Bax, the ratio of Bcl-2 to Bax. Data are expressed as mean ± standard error (n = 6). * *p* < 0.05, ** *p* < 0.01.

As shown in Figure 6, PQ challenge significantly increased the activities of caspase 3 and caspase 9 in the liver, which was reversed by silybin supplementation (*p* < 0.05) (Figure 5A,B). Interestingly, dietary silybin tended to decrease the activity of caspase 3 and caspase 9 in the non-challenged piglets (*p* = 0.07 and *p* = 0.09, respectively). Western blot analysis showed that PQ challenge significantly enhanced the protein expression of the Cleaved caspase 3 in the liver compared with the control group (*p* < 0.01) (Figure 5D). Conversely, silybin supplementation markedly downregulated Cleaved caspase 3 protein expression, upregulated B-cell lymphoma-2 (Bcl-2) protein expression, and increased the ratio of Bcl-2 to Bcl-2-associated-X-protein (Bax) in piglets upon PQ challenge (*p* < 0.05) (Figure 5D,E,G). Furthermore, silybin also increased Bcl-2 protein expression in the non-challenged piglets (*p* < 0.01) (Figure 5E). There was no significant difference in the Bax protein expression among all groups (*p* > 0.05) (Figure 5F).

## 4. Discussion

The liver is a highly metabolically active organ in mammals that plays an important role in maintaining systemic health but is extremely susceptible to oxidative stress caused by various factors. Herein, we investigated the protective effect of silybin on liver injury induced by PQ challenge in weaned piglets and its potential molecular mechanism. Consistent with previous reports [30,31,32], histopathological staining analyses showed that PQ challenge caused noticeable pathological changes in the liver, evidenced by hepatic karyopyknosis, karyolysis, and vacuolation of hepatocytes. However, silybin supplementation alleviated hepatic morphological injury induced by PQ in piglets, which was proved by a reduced pathological score. A previous study also suggested that silymarin administration mitigated fibrosis, granulomatosis, and cytolytic necrosis in the liver of CCl_4_-challenged broiler chickens [33]. Plasma biochemical indices such as AST, ALT, and ALP are the most sensitive markers of the degree of liver damage, and the increased activity of these enzymes in plasma indicates increased liver damage [34]. In the present study, dietary silybin supplementation lowered plasma ALT and AST activity in the PQ-challenged piglets, suggesting that silybin may have protective effects against liver injury induced by PQ treatment. These results were in agreement with those observed by Zhang et al. (2021) [35], which verified that micelle silymarin supplementation significantly decreased serum AST activity in sows on day 21 postpartum. In addition, silybin addition decreased the serum activities of ALT and γ-glutamyl transpeptidase in Peking ducks [18]. Taken together, these results indicate that dietary silybin administration has a protective function against the hepatic injury induced by PQ in piglets.

It is known that maintaining a proper redox balance is an effective strategy for alleviating liver injury [36]. The antioxidant enzymes secreted by hepatocytes are the primary line of defense against oxidative stress. In particular, SOD is an essential component of the antioxidant enzyme system, catalyzing the disproportionation of superoxide anion radicals into oxygen and hydrogen peroxide; the latter is further decomposed into water and oxygen by CAT [37]. In addition, GSH-Px also has a strong ability to scavenge free radicals, and T-AOC can reflect the overall antioxidant capacity of the body [38,39]. A previous study suggested that PQ challenge triggered severe oxidative stress in piglets [25]. Consistent with this result, our data also showed that PQ treatment decreased GSH-Px activity and T-AOC levels and augmented MDA and H_2_O_2_ levels in the liver. More importantly, dietary silybin supplementation has been shown to enhance SOD and GSH-Px activity and T-AOC levels, and subsequently lower H_2_O_2_ content and the level of lipid peroxidation. Another study also corroborated that silymarin addition protected against oxidative stress in gilts by reducing protein carbonyl contents in hepatic tissue [40]. Jiang et al. (2020) [15] reported that silymarin supplementation alleviated oxidative stress caused by pregnancy through enhancing serum activities of CAT and GSH-Px in sows. These results indicated that dietary silybin supplementation relieved oxidative stress by regulating the function of the antioxidant defense system.

As mentioned above, the hepatoprotective effects of silybin may be intimately related to its regulation of antioxidant capacity. Thus, the effect of silybin on the activation of the Nrf2 signaling pathway in the liver was further investigated. Nrf2/Keap1 signaling plays a vital role in resisting oxidative injury. Impaired oxidative redox status induces the dissociation of Nrf2 from Keap1 and translocation into the nucleus, which then binds to the antioxidant response element to regulate the expression of downstream antioxidant genes, such as CAT, SOD, and GPx, thus coping with cells from oxidative-stress-induced cellular injury [41,42]. In the present study, we found that Nrf2 protein expression significantly increased in response to silybin supplementation, and the mRNA expression of *SOD1*, *GPX1*, and *GPX4* was also in parallel with the alteration trend of Nrf2 protein expression, suggesting that dietary silybin enhanced antioxidant capacity in the PQ-challenged piglets. Coherently, our previous research found that dietary silybin improved intestinal antioxidant capacity in weaned piglets through regulating the Nrf2 signaling pathway [19]. Another study indicated that silymarin administration alleviated thioacetamide-induced acute liver injury by activating the Nrf2/Keap1 pathway [43]. An in vitro study also confirmed that silybin addition attenuated H_2_O_2_-induced oxidative stress by modulating Nrf2 signaling [44]. Taken together, these results demonstrated that dietary silybin supplementation mitigated liver damage through regulating the Nrf2 signaling pathway and subsequently enhancing antioxidant capacity in piglets.

Oxidative stress can trigger an inflammatory response, which in turn can directly exacerbate redox imbalance [45]. The increased content of proinflammatory cytokines is tightly related to the activated inflammatory response [46]. In the present study, PQ challenge elevated the secretion of TNF-α and IL-8 and their gene expression in the liver compared with the control group. However, dietary silybin dramatically reversed these trends and increased IL-10 concentration in the PQ-challenged piglets, which indicated that silybin attenuated the PQ-induced excessive inflammatory response by diminishing the generation of proinflammatory cytokines. Consistent with our results, dietary silybin dose-dependently ameliorated triptolide-induced liver injury in rats by suppressing proinflammatory cytokine TNF-α, IL-6, and IL-1β production [47]. Similarly, silymarin supplementation decreased serum TNF-α and IL-1β concentration in lactating sows [15,48]. It is well known that the NF-κB signaling pathway plays an important role in inflammatory responses, which can promote the expression of downstream target genes and the secretion of inflammatory mediators when activated by external stimuli [49,50]. A previous study has observed that PQ challenge triggers an inflammatory response by elevating the phosphorylation of NF-κB in the liver of weaned piglets [25]. In the current study, increased protein abundance of P-NF-κB and NF-κB was also found after PQ treatment, but dietary silybin significantly suppressed the protein expression of P-NF-κB and NF-κB compared with PQ-challenged piglets. The passivation of the NF-κB signaling further reduced the expression of proinflammatory cytokines, which was consistent with the alteration trend of cytokine levels in the liver. In line with our results, some studies have also reported that silybin inhibited the NF-κB signaling cascade to alleviate hepatic inflammation and steatohepatitis [51,52]. Taken together, these results indicated that dietary silybin supplementation mitigated hepatic inflammation signaling by abrogating the activation of the NF-κB pathway to reduce the expression of inflammatory cytokines.

The liver contains a large number of mitochondria, which play an essential role in cellular bioenergetics, regulating redox balance and apoptosis [53]. The mitochondrial respiratory chain consists of five enzymatic complexes, which are mainly responsible for the generation of ATP through oxidative phosphorylation [54]. Oxidative stress can cause mitochondrial dysfunction by disturbing cellular bioenergetics [55]. Herein, we found that PQ-induced oxidative stress reduced the activities of mitochondrial complexes I and V and lowered the mRNA expression level of *NDUFV2* (complex I) and *ATP5H* (complex V) in the liver. However, silybin supplementation alleviated the deleterious effects of PQ and normalized abnormal expression of the respiratory chain genes and subsequently recovered mitochondrial complex activity in the liver of the piglets, thereby enhancing the production of ATP. This is consistent with our previous research, which suggested that dietary silybin improved mitochondria function by increasing mitochondrial complex activity and ATP content in the intestine of weaned piglets [19]. In addition, another study also showed that silybin improved liver injury induced by NAFLD in mice by restoring hepatic mitochondrial respiratory chain activity (for all five complexes) [13]. The maintenance of mitochondrial function requires a proper balance between fission and fusion processes [56]. In this study, we found that PQ challenge increased the expression level of the mitochondrial fission-associated protein Drp1 and decreased the expression level of the mitochondrial fusion-associated protein Mfn2, implying that PQ treatment disrupted the balance of mitochondrial fission and fusion. Conversely, silybin supplementation markedly normalizes mitochondrial dynamic imbalance under PQ challenge. An in vitro study also suggested that silybin treatment protects human neuroblastoma SH-SY5Y cells from H_2_O_2_-induced mitochondrial damage by regulating OPA1 and Drp1 protein expression [57]. Collectively, silybin supplementation protected against PQ-induced liver injury in piglets by improving mitochondrial functions.

Previous research has demonstrated that mitochondrial dysfunction can exacerbate oxidative stress and trigger cell apoptosis, ultimately leading to liver injury and dysfunction [58]. Caspase 3 is an essential executioner in the process of apoptosis that can be heterologous activated by caspase 9 [59]. Herein, we found that dietary silybin inhibited PQ-triggered apoptosis by antagonizing an increase in caspase 3 and caspase 9 activity, which is in keeping with previous studies showing that pretreatment with silybin decreased the percentage of caspase-3-positive cells induced by H_2_O_2_ and silybin supplementation inhibited the increase in intestinal caspase 3 and caspase 9 activity induced by PQ challenge [19,60]. Interestingly, dietary silybin also decreased caspase 3 and 9 activity in the liver of non-challenged piglets. These results indicated that silybin has promising anti-apoptotic capacity. Among members of the Bcl-2 family, Bcl-2 and Bax proteins play an essential role in regulating cell apoptosis, and the former has anti-apoptotic effects, while the latter has the opposite [61]. In this study, silybin supplementation prevented PQ-induced cell apoptosis by enhancing the expression of anti-apoptotic proteins such as Bcl-2 and the ratio of Bcl-2 to Bax and inhibiting the expression of the proapoptotic protein Cleaved caspase 3, which may further clarify the underlying mechanism by which dietary silybin alleviated liver injury induced by PQ. Similar results were also found in both in vivo and in vitro models [62,63]. Overall, the results suggested that silybin supplementation suppressed PQ-induced hepatocyte apoptosis in piglets by regulating the Bcl-2/Bax signaling pathway.

## 5. Conclusions

Our study demonstrated that dietary silybin supplementation mitigated hepatic injury from oxidative stress induced by PQ in weaned piglets, which is closely associated with enhancing antioxidant capacity, mitigating inflammation, improving mitochondrial function, and inhibiting apoptosis. These findings provide a theoretical basis for applying silybin as an antioxidant in animal feed and improving liver health through dietary interventions.

## Figures and Tables

**Table 1:** The primers for quantitative real-time PCR.

Gene ^1^	Accession Number	Primer Sequence (5′-3′)	Product Size (bp)
*GAPDH*	NM_001206359.1	F: GCTTGTCATCAATGGAAAGG	86
		R: CATACGTAGCACCAGCATCA	
*TNF-α*	NM_214022.1	F: CTCACGTCCTTCTGGTTTAG	96
		R: CCCTGATTTCTAAGTGTTGC	
*IL-6*	NM_214399.1	F: AATGTCGAGGCTGTGCAGATT	82
		R: TGGTGGCTTTGTCTGGATTCT	
*IL-8*	NM_213867.1	F: CCGTGTCAACATGACTTCCAA	75
		R: GCCTCACAGAGAGCTGCAGAA	
*IL-10*	NM_214041.1	F: GACGATGAAGATGAGGAAGA	54
		R: AGGTTTTTCTTTGGTTTCCC	
*CAT*	NM_214301.2	F: CCTGCAACGTTCTGTAAGGC	72
		R: GCTTCATCTGGTCACTGGCT	
*SOD1*	NM_001190422.1	F: GAAGACAGTGTTAGTAACGG	93
		R: CAGCCTTGTGTATTATCTCC	
*GPX1*	NM_214201.1	F: TCTCCAGTGTGTCGCAATGA	104
		R: TCGATGGTCAGAAAGCGACG	
*GPX4*	NM_214407.1	F: GATTCTGGCCTTCCCTTGC	173
		R: TCCCCTTGGGCTGGACTTT	
*NDUFS2*	XM_005663166.3	F: CTAAACGCGCAGAGATGAAGA	108
		R: CCTCAATGGCAGTGTATGTGG	
*NDUFV2*	NM_001097475.2	F: CCCAGATACTCCATTTGATTTCA	169
		R: AATTTCTGCCACCTTGTTCATG	
*SDHA*	XM_021076930.1	F: TCTCTGAGGCCGGGTTTAACACA	124
		R: CACCTCCAGTTGTCCTCCTCCAT	
*UQCRB*	NM_001185172.1	F: GGATGACGATGTAAAAGAAGCCA	141
		R: TCCTCCTCATATTTTGTCCACTG	
*ATP5H*	XM_021066093.1	F: CATTGACTGGGTAGCCTTTG	115
		R: CTTCTCAGGTAGAGCAGCCA	

^1^ *GAPDH*, glyceraldehyde-3-phosphate dehydrogenase; *TNF-α*, tumor necrosis factor-α; *IL*, interleukin; *CAT*, catalase; *SOD*, superoxide dismutase; *GPX*, glutathione peroxidase; *NDUFS2*, NADH ubiquinone oxidoreductase core subunit S2; *NDUFV2*, NADH ubiquinone oxidoreductase core subunit V2; *SDHA*, succinate dehydrogenase complex flavoprotein subunit A; *UQCRB*, ubiquinol-cytochrome c reductase binding protein; *ATP5H*, ATP synthase subunit d.

## Data Availability

The datasets of the current study are available.
